# Metabolomic Profiling and Genomic Study of a Marine Sponge-Associated *Streptomyces* sp

**DOI:** 10.3390/md12063323

**Published:** 2014-06-02

**Authors:** Christina Viegelmann, Lekha Menon Margassery, Jonathan Kennedy, Tong Zhang, Ciarán O’Brien, Fergal O’Gara, John P. Morrissey, Alan D. W. Dobson, RuAngelie Edrada-Ebel

**Affiliations:** 1 Strathclyde Institute of Pharmacy and Biomedical Sciences, University of Strathclyde, The John Arbuthnott Building, 161 Cathedral Street, Glasgow, Scotland G4 0RE, UK; E-Mails: christina.viegelmann@strath.ac.uk (C.V.); tong.zhang.101@strath.ac.uk (T.Z.); 2 Marine Biotechnology Centre, Environmental Research Institute, University College Cork, Lee Road, Cork, Ireland; E-Mails: lekha513@gmail.com (L.M.M.); sarklor@gmail.com (C.O.); f.ogara@ucc.ie (F.O.); j.morrissey@ucc.ie (J.P.M.); a.dobson@ucc.ie (A.D.W.D.); 3 School of Microbiology, University College Cork, Cork, Ireland; 4 Biomerit Research Centre, University College Cork, Cork, Ireland; 5 School of Biomedical Sciences, Curtin University, Perth 6102, WA, Australia

**Keywords:** *Streptomyces*, *Haliclona simulans*, metabolomics, antimycin, antifungal, butenolide

## Abstract

Metabolomics and genomics are two complementary platforms for analyzing an organism as they provide information on the phenotype and genotype, respectively. These two techniques were applied in the dereplication and identification of bioactive compounds from a *Streptomyces* sp. (SM8) isolated from the sponge *Haliclona simulans* from Irish waters. *Streptomyces* strain SM8 extracts showed antibacterial and antifungal activity. NMR analysis of the active fractions proved that hydroxylated saturated fatty acids were the major components present in the antibacterial fractions. Antimycin compounds were initially putatively identified in the antifungal fractions using LC-Orbitrap. Their presence was later confirmed by comparison to a standard. Genomic analysis of *Streptomyces* sp. SM8 revealed the presence of multiple secondary metabolism gene clusters, including a gene cluster for the biosynthesis of the antifungal antimycin family of compounds. The antimycin gene cluster of *Streptomyces* sp. SM8 was inactivated by disruption of the antimycin biosynthesis gene *ant*C. Extracts from this mutant strain showed loss of antimycin production and significantly less antifungal activity than the wild-type strain. Three butenolides, 4,10-dihydroxy-10-methyl-dodec-2-en-1,4-olide (**1**), 4,11-dihydroxy-10-methyl-dodec-2-en-1,4-olide (**2**), and 4-hydroxy-10-methyl-11-oxo-dodec-2-en-1,4-olide (**3**) that had previously been reported from marine *Streptomyces* species were also isolated from SM8. Comparison of the extracts of *Streptomyces* strain SM8 and its host sponge, *H. simulans*, using LC-Orbitrap revealed the presence of metabolites common to both extracts, providing direct evidence linking sponge metabolites to a specific microbial symbiont.

## 1. Introduction

Marine sponges are at the forefront of marine natural product discovery. Being sessile animals, they rely mainly on chemical means of defense. Sponges produced 37% of all novel marine natural products discovered in 2002 [1]. The latest review showed that sponges accounted for 28.6% of novel compound discoveries from marine sources in 2012, excluding those isolated from sponge-associated microorganisms [2].

Sponges play host to a wide variety of endosymbiotic microorganisms such as bacteria, fungi, yeast, dinoflagellates, diatoms and microalgae [3]. Indeed, some of the compounds previously isolated from sponges have been proven to be products of their endosymbionts [4,5,6,7]. This is a distinct advantage in the search for new drugs as this bypasses the supply problem created by the difficulties in procuring sufficient quantities of the desired compounds from marine macroorganisms. In addition, the genome of the microorganism, as well as the culture conditions, can be manipulated to optimize the production of the preferred metabolite.

The bacterial communities of *Haliclona* sponges from across the globe have been studied [8,9,10,11] and these show a diverse range of endosymbionts, many of which produce bioactive metabolites. Of the 52 bacteria isolated from *H. simulans* from the Irish Sea, 29 possessed antibiotic activity against at least one of the bacterial or fungal test strains [8].

**Figure 1:**
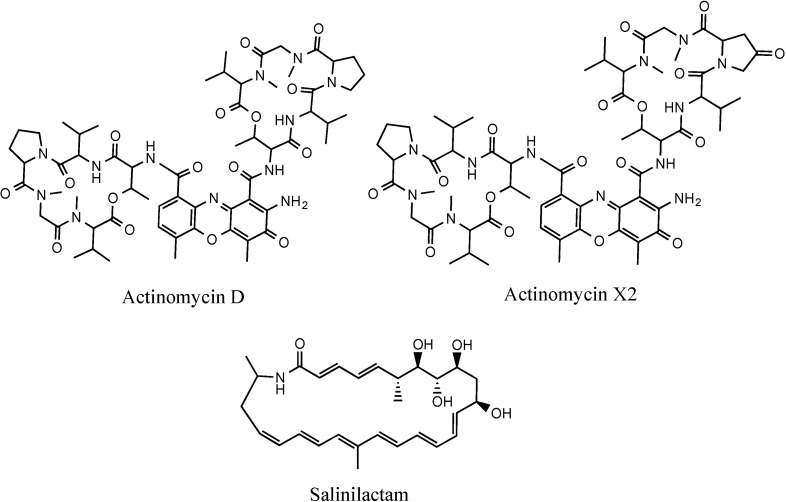
Structures of actinomycin D, actinomycin X2 and salinilactam, isolated from strains of *Streptomyces*.

A *Streptomyces* sp., SM8, was isolated from *H. simulans* collected from Irish waters. Partial 16S rRNA sequencing indicated that this strain bore 100% similarity to *Streptomyces violascens* XSD-115 and several other *Streptomyces* species [8]. Other strains of *S. violascens* have been reported to produce antibiotics such as actinomycin D and actinomycin X_2_ (V) [12] (Figure 1), protease inhibitors such as trypsin and chymotrypsin inhibitors [13], and 3β-hydroxysteroid oxidase [14].

Metabolomics and genomics are two complementary platforms in systems biology. Genomics details the genotype of the organism whereas metabolomics reveals its phenotype. While the former is widely established, the latter is a relatively new field that is still evolving as both technology and various methodologies are improved and refined [15,16]. Although both platforms are used in natural product discovery, relatively few studies have used both approaches. Seipke *et al.* used a combination of genomics and chemical isolation to characterize antifungal compounds from actinomycetes [17,18] and genomic and chemical techniques have also been used to study *Salinispora tropica*, a marine actinomycete, leading to the structural elucidation of salinilactam A and providing a greater depth of information on the biosynthetic capabilities of *S. tropica* [19]. The dual approach of metabolomics and genomics was used to analyze SM8 in order to facilitate the identification and isolation of bioactive compounds with a particular focus on antifungal metabolites.

Metabolomic techniques such as nuclear magnetic resonance (NMR) spectroscopy and liquid chromatography-high resolution mass spectrometry (LC-HRMS) were applied in the preliminary screening of metabolites produced by *Streptomyces* strain SM8. These two complementary analytical techniques allow the rapid identification of classes of compounds present in the samples as well as the putative identification of specific compounds [20,21]. However, although LC-HRMS is extremely sensitive and can detect compounds present in very low quantities, there are certain classes of compounds that cannot be detected by the mass spectrometer, the reason being that they are unable to be ionized. NMR, on the other hand, has no separation step and therefore provides a snapshot of the metabolome of the sample. It is less sensitive than MS but is more reproducible and has no discrimination in detection depending on the concentration of the sample and the power of the spectrometer. Both methods of analysis can be applied in the structural elucidation of compounds.

Extracts of *Streptomyces* strain SM8 possessed antifungal and antibacterial activity. The goal of this study was to use the dual approach of metabolomics and genomics to analyze *Streptomyces* strain SM8 in order to facilitate the identification and isolation of bioactive compounds with a particular focus on antifungal metabolites. Metabolomics was further applied in the comparison of the extract of *Streptomyces* strain SM8 with that of its host sponge to determine whether compounds produced by the bacteria could be found in the sponge.

## 2. Results and Discussion

*Streptomyces* strain SM8, isolated from the sponge *H. simulans*, was cultured on two types of media: oatmeal media (OM-SW) and starch-yeast extract-peptone (SYP-SW) media. The crude extracts, prepared by solid phase extraction with Amberlite^®^ XAD-16 resin (Rohm and Haas Company, Philadelphia, PA, USA), exhibited activity against *Bacillus subtilis*, *Pseudomonas aeruginosa*, *Candida albicans*, *C. glabrata*, *Saccharomyces cerevisiae*, *Aspergillus fumigatus* and *Kluyveromyces marxianus*.

### 2.1. Genomic Analysis of Streptomyces Strain SM8

The genome sequence of *Streptomyces* strain SM8 was determined by Roche 454 pyrosequencing. Following assembly, the draft genomic sequence (Table 1) consisted of 534 contigs with a total size of 7.2 Mb in and a GC content of 73%. A total of 6647 protein coding genes were annotated, including 31 putative non-ribosomal peptide synthetase (NRPS) modules and 25 polyketide synthase (PKS) modules were identified in the draft genome in addition to other genes predicted to be involved in the biosynthesis of secondary metabolites. The genome sequence is deposited at GenBank with accession number PRJNA180938.

**Table 1:** Genomic data for *Streptomyces* strain SM8.

Genomic data for *Streptomyces* strain SM8	
Genome size	7.155 Mb
Contigs	534
GC content	73%
Gene count	6722
Protein coding genes	6647
RNA genes	75
NRPS modules	31
PKS modules	25

**Figure 2:**
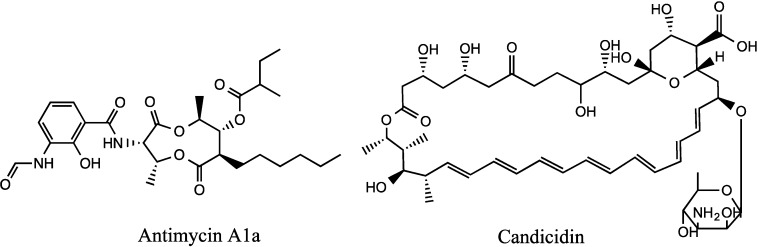
Structures of antimycin A1 and candicidin.

Secondary metabolism gene clusters for the known antifungal metabolites antimycin and candicidin (Figure 2) were identified in the *Streptomyces* SM8 genome. The putative candicidin gene cluster was spread over 18 contigs in the assembly, however analysis of the cluster by comparison to known polyene PKS clusters confirmed that all the predicted biosynthesis genes were present. The antimycin gene cluster was also found to be intact by comparison to the published cluster from *Streptomyces albus* sp. S4 [18]. Other predicted secondary metabolism gene clusters present in the *Streptomyces* SM8 genome include a large NRPS gene cluster, similar to a linear gramicidin biosynthesis cluster, encoding a predicted 16 amino acid linear peptide and additional smaller NRPS, PKS and hybrid secondary metabolism gene clusters. In addition to the gene clusters for modular PKS and NRPS the genome was also found to contain genes and gene clusters predicted to be involved in the biosynthesis of terpenes, ribosomally encoded peptide antibiotics and siderophores.

### 2.2. Identification of Bioactive Metabolites from Streptomyces Strain SM8

#### 2.2.1. Chemical Analysis of Antibacterial and Antifungal Fractions from Small-Scale SM8 Extract

Crude extract, prepared by solid phase extraction with Amberlite^®^ XAD-16 resin (Rohm and Haas Company, Philadelphia, PA, USA), from batch cultures of *Streptomyces* strain SM8 was fractionated with water and methanol using reverse phase medium pressure liquid chromatography (MPLC). Fractions were assessed for antimicrobial activities against *C. albicans* and *B. subtilis*, the fungal and bacterial strains that were most sensitive to the crude extract. The minimum inhibitory concentrations (MICs) of the most active fractions are shown in Table 2.

**Table 2:** MICs of the most active fractions from the SM8 extract.

Pooled Fraction	Yield (mg)	*B. subtilis* MIC (μg·mL^−1^)	*C. albicans* MIC (μg·mL^−1^)
221–226	6.5	7.42	240
227–230	9.5	9.40	210
239–242	9.1	400	80
243–248	13.0	400	90

**Figure 3:**
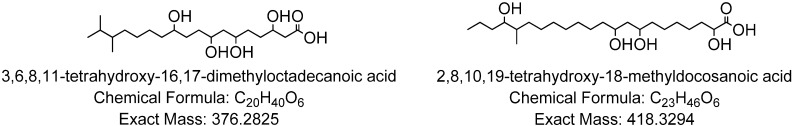
Structure of polyhydroxylated saturated fatty acids found in fractions 221–230.

**Figure 4:**
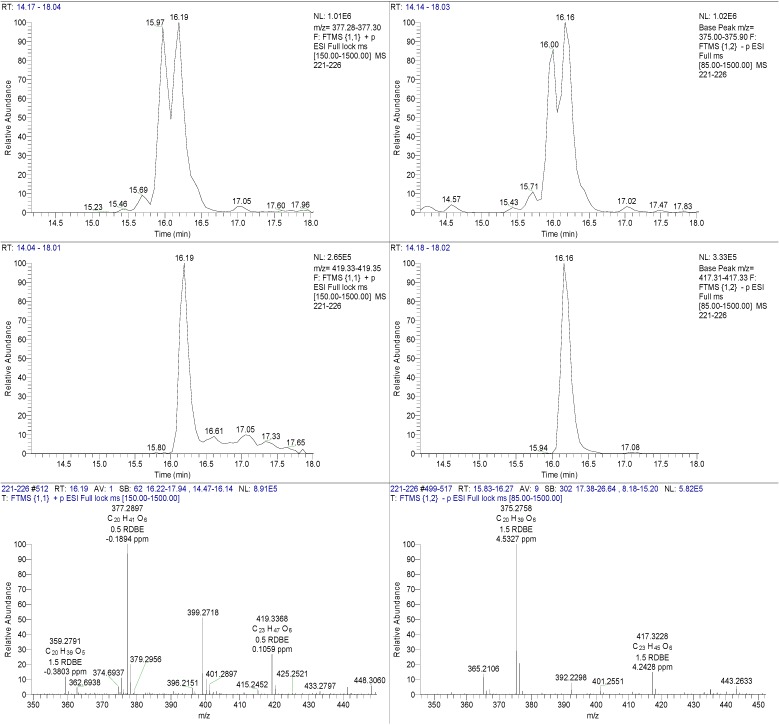
Extracted ion chromatograms and high resolution mass spectra of fractions 221–230.

**Figure 5:**
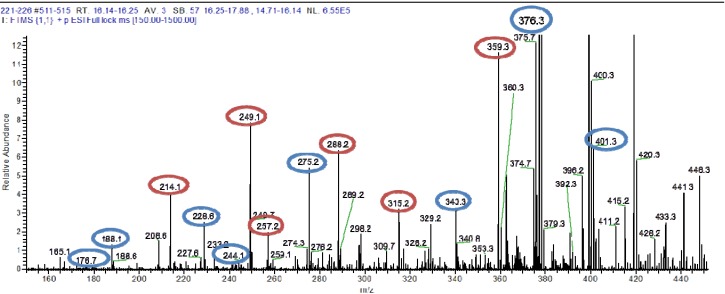
MS*^n^* fragmentation of the polyhydroxylated lipids found in fractions 221–230. Source fragmentation data in the positive mode summarizing the MS*^n^* ion peaks for *m/z* 377.2898 (red) and 419.3668 (blue).

**Figure 6:**
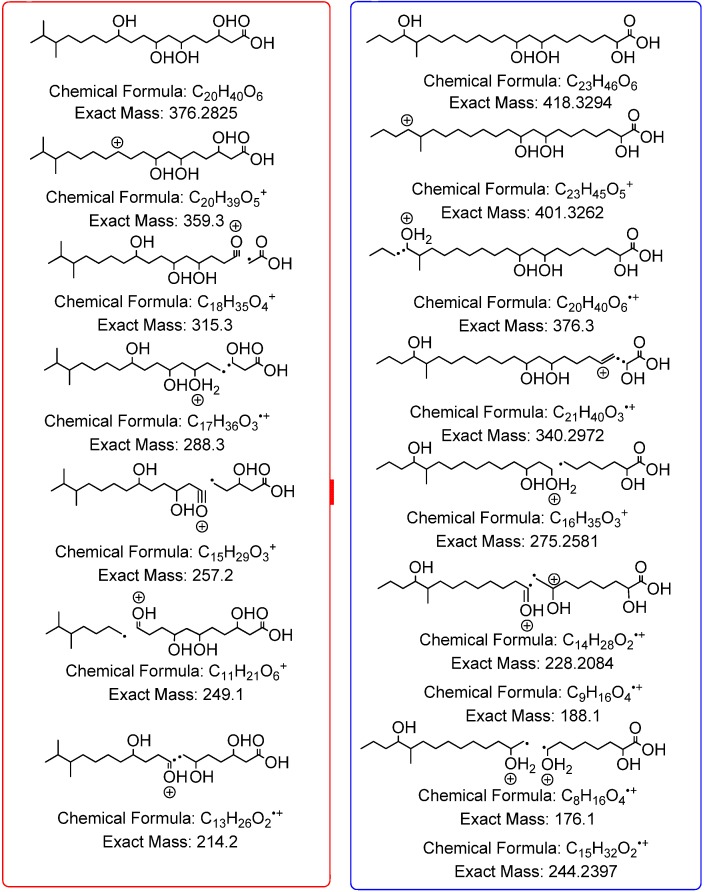
Proposed fragmentation pathway for 3,6,8,11-tetrahydroxy-16,17-dimethyloctadecanoic acid (C_20_H_40_O_6_) and 2,8,10,19-tetrahydroxy-18-methyldocosanoic acid (C_23_H_46_O_6_).

**Figure 7:**
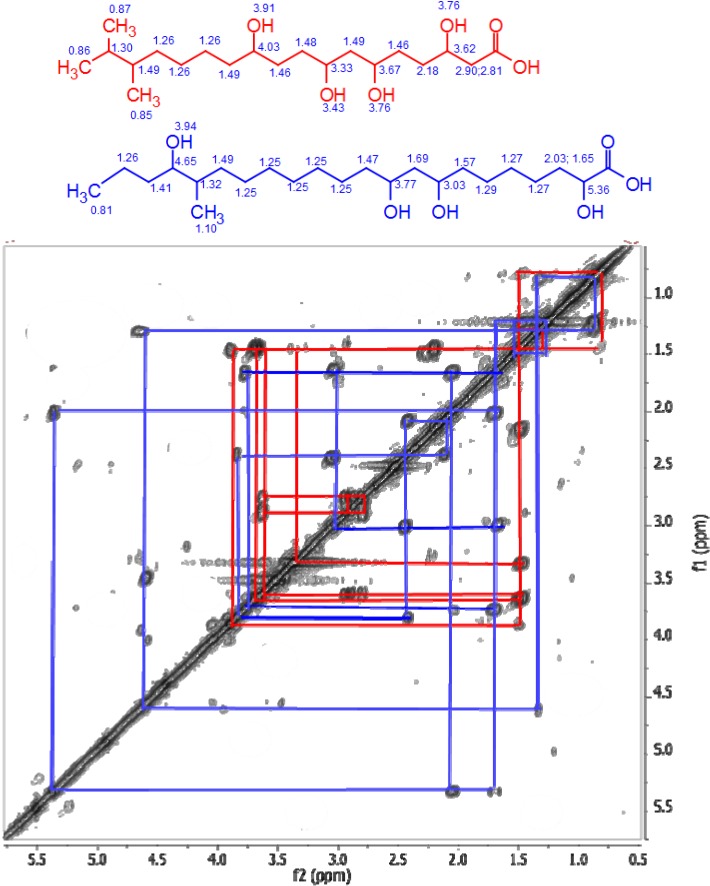
^1^H-COSY NMR of fractions 221–226 and 227–230, which were the most potent against *B. subtilis*. (Full spectral data in Supplementary Information).

**Figure 8:**
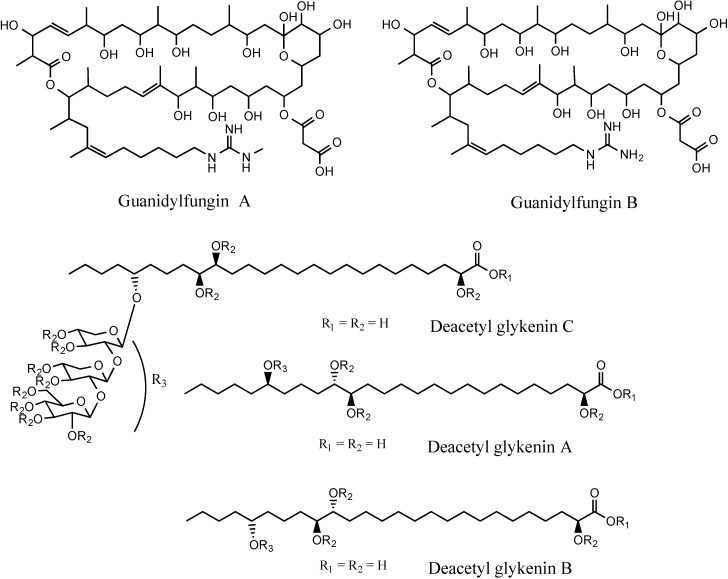
Structures of the guanidylfungins and deacetyl glykenins.

HRESIMS data of the semi-purified fractions 221–230 revealed the occurrence of two major inseparable components with ion peaks at *m/z* 375.2757/417.3228 [M − H]^−^ in negative mode and *m/z* 377.2898/419.3668 [M − H]^+^ in positive mode with the molecular formulas of C_20_H_40_O_6_ and C_23_H_46_O_6_, respectively. Both ion peaks designated with RDB of 1.0 indicated the aliphatic and polyhydroxylated lipid nature of the structure. The ^1^H NMR spectrum in DMSO-*d*_6_ exhibited that the major compounds in the antibacterial fractions were indeed polyhydroxylated saturated fatty acids. Peaks belonging to terminal methyl groups (δ_H_ 0.80 to 1.00), aliphatic C*H_2_* groups (δ_H_ 1.24 to 1.30), hydroxyl-bound C*H* (δ_H_ 3.00 and 4.50), and the C*H*_2_ group attached to the acid terminus (δ_H_ 2.19 to 2.80) are characteristic of polyhydroxylated saturated fatty acids. The oxymethine protons also corresponded to peaks resonating between 60 and 75 ppm as shown by its ^13^C NMR spectrum (see Supplementary Information). The presence of two polyhydroxylated saturated fatty acid congeners (Figure 3) at a ratio of 1:1 was confirmed by their extracted ion chromatograms (Figure 4). Due to the overlapping carbon resonances of the oxymethine, long chain methylene and terminal methyl groups, it was not possible to deduce unambiguously the position of the hydroxyl groups by HMBC. MS*^n^* fragmentation in the positive mode revealed the position of the hydroxyl units on the alkyl chain through homolytic bond cleavage as shown in Figure 5 and Figure 6. This was then unambiguously supported by the COSY NMR data (Figure 7). The two polyhydroxylated saturated fatty acids were then elucidated as 3,6,8,11-tetrahydroxy-16,17-dimethyloctadecanoic acid and 2,8,10,19-tetrahydroxy-18-methyldocosanoic acid, respectively. When compared to the *ca.* 3–4 ppm proton chemical shifts of oxymethine hydrogens, the proton resonance of the terminal 2-hydroxybutanoic acid moiety was deshielded to 4–5 pm which was comparable to those of the glykenin antibiotics (Figure 8) [22].

Aside from *Streptomyces* strain SM8, other species of *Streptomyces* such as *S. scabies* primarily produce saturated fatty acids rather than unsaturated fatty acids [23]. Hydroxy saturated fatty acids have previously been reported to have activity against some strains of fungi [24], whereas other saturated fatty acids have been proven to inhibit the growth of methicillin-resistant *Staphylococcus aureus* [25]. Polyhydroxy saturated fatty acids have previously been isolated from fungi such as *Ulocladium botrytis* and *Haematomma ventosum* [26,27]. They have also been found as degradation products of guanidylfungin A and B (Figure 8), metabolites of *S. hygroscopicus* that are active against fungi and Gram-negative bacteria [28,29]. The tetrahydroxy saturated fatty acids also comprise the aglycone portion of the deacetyl glykenins A–C (Figure 8) which are glycosidic antibiotics produced by *Basidiomycetes* sp. [22].

The active fractions were also analyzed by LC-Orbitrap and based on accurate mass (±3 ppm), we searched the Dictionary of Natural Products (DNP) database to putatively identify metabolites that had previously been reported as products of *Streptomyces* sp. As seen in Table 3, the predominant identified compounds were members of the antimycin family.

**Table 3:** *Streptomyces* metabolites dereplicated from active fractions using the DNP 2012 database. Metabolites in this table are limited to those with the largest peak area and from the biological source *Streptomyces* sp. Kitamycin also belongs to the antimycin family of antifungal compounds.

Fraction		*m/z*	RT (min)	Name	Formula
221–226	[M + H]^+^	465.2235	19.53	Kitamycin A or B	C_23_H_32_N_2_O_8_
	[M − H]^−^	519.2353	19.43	Antimycin A3 or A7	C_26_H_36_N_2_O_9_
227–230	[M + H]^+^	535.2652	20.70	Antimycin A2, A8, A11 or A17	C_27_H_38_N_2_O_9_
	[M − H]^−^	533.2512	20.67		
239–242	[M + H]^+^	521.2493	24.34	Antimycin A3 or A7	C_26_H_36_N_2_O_9_
	[M − H]^−^	519.2352	24.25		
243–248	[M + H]^+^	535.2650	25.63	Antimycin A2, A8, A11 or A17	C_27_H_38_N_2_O_9_
	[M − H]^−^	519.2352	24.25	Antimycin A3 or A7	C_26_H_36_N_2_O_9_

#### 2.2.2. Deletion of Antimycin Gene Cluster from *Streptomyces* Strain SM8

**Figure 9:**
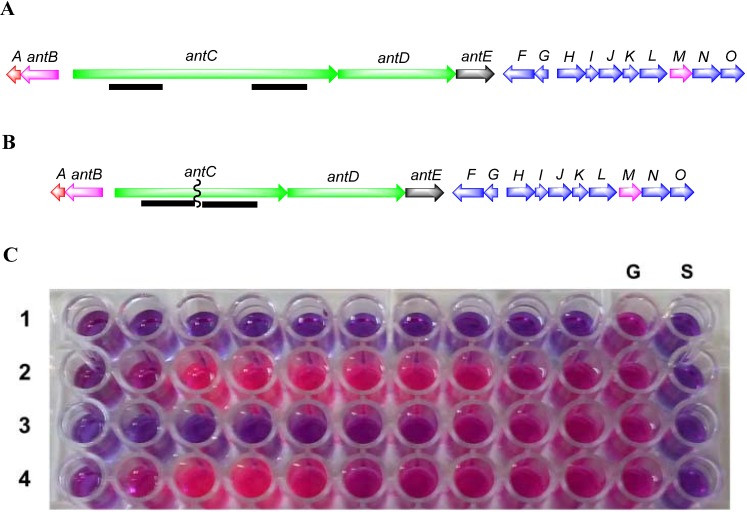
Disruption of antimycin biosynthesis cluster in *Streptomyces* strain SM8 by deletion of *ant*C. Panel **A**: Characterized antimycin gene cluster containing genes *ant*A to *ant*O. Genes are color coded according to predicted roles in pathway; regulation—red; NRPS and PKS scaffold biosynthesis—green; FSA starter unit biosynthesis—blue; post-PKS/NRPS tailoring—pink; PKS extender unit supply—grey. The *ant*C gene encodes an NRPS gene essential for antimycin biosynthesis. To disrupt the antimycin gene cluster regions from upstream and downstream of the *ant*C gene (shown underlined) were PCR amplified and cloned into the vector pKC1139 to produce pKC1139A1A2. This plasmid was introduced into *Streptomyces* strain SM8 in order to delete the *ant*C gene as described; Panel **B**: antimycin gene cluster following deletion of a 3 kb part of the *ant*C gene; Panel **C**: Antifungal bioassay of *ant*C mutant strain. Antifungal activities of a dilution series of extracts (rows 1–4) were determined against *C. albicans*; 1—WT extract grown in OM-SW broth; 2—Δ*ant*C extract grown in OM-SW broth; 3—WT extract from SYP-SW broth; 4—Δ*ant*C extract from SYP-SW; column G—no extract; column S—no inoculum.

Genomic analysis of *Streptomyces* strain SM8 revealed the presence of an antimycin biosynthetic gene cluster with high similarity to and the same gene organization as that identified from *Streptomyces albus* S4 [18]. In order to determine the contribution of antimycin to the total antifungal activity of the strain this gene cluster was inactivated. The *ant*C gene, encoding a NRPS central to the antimycin biosynthesis pathway, was deleted from the strain (Figure 9). The deletion in the resulting strain, SM8*-*Δ*ant*C, was confirmed by PCR.

**Figure 10:**
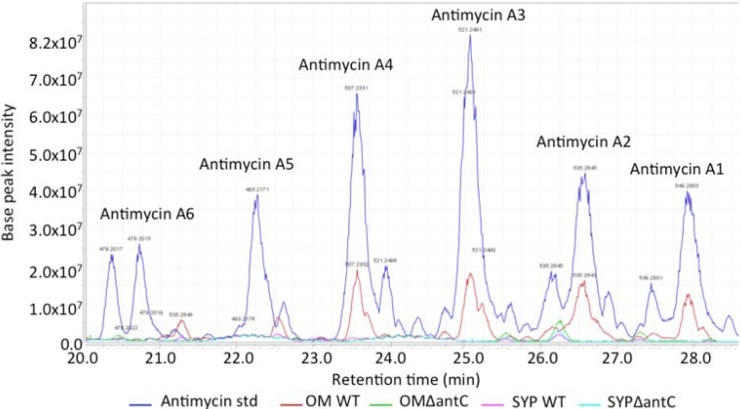
Analyses of antimycin production in wild-type and mutant strains. Extracts were analyzed by LC-Orbitrap and compared to the antimycin A standard. Although the standard is alleged to contain antimycin A1 to A4, peaks corresponding to antimycin A5 and A6 were also identified. The samples are as follows: antimycin A standard (dark blue); extract from SM8 WT grown in OM-SW (red); extract from SM8*-*Δ*ant*C mutant grown in OM-SW (green); extract from SM8 WT grown in SYP-SW (pink); extract from SM8*-*Δ*ant*C mutant grown in SYP-SW (light blue).

**Figure 11:**
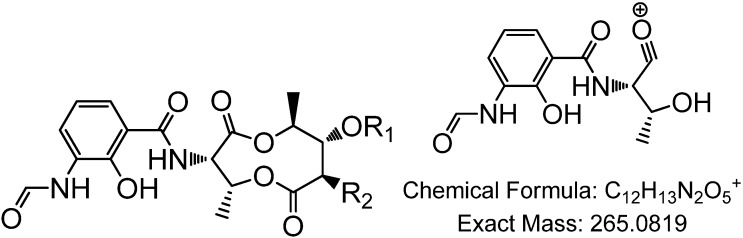
Antimycins A1 to A4. The fragmentation of the ring to generate the 265 [M + H]^+^ ion, common to antimycins A1a/b to A4a/b, is also shown.

Both the mutant (SM8*-*Δ*ant*C) and wild-type SM8 were cultured on OM-SW and SYP-SW and the resulting extracts were subjected to bioassays. Extracts from the SM8*-*Δ*ant*C mutant strain were shown to have ~32-fold less antifungal activity than the wild-type SM8 (Figure 9). LC-Orbitrap analysis of the culture extracts in comparison to an antimycin A standard mixture (Sigma, St. Louis, MO, USA) confirmed the presence of antimycins A1, A2, A3 and A4 in the wild-type but not in the mutant SM8 strain (Figure 10). Although the datasheet of Sigma-Aldrich (St. Louis, MO, USA) claimed it only contains antimycins A1–A4, A5 and A6 were also seen in the LC-MS. The OM-WT extract contained compounds with the same *m*/*z* and retention time as the standard, as evidenced in Figure 10, in addition to other compounds that had the same *m*/*z* but different retention times. It is likely that these are additional isomers of antimycin, not present in the standard, as the majority of these contain the fragment ion at *m/z* 265.081 [M + H]^+^, which is characteristic of the antimycins. The SYP-WT extract did not contain any antimycin compounds that were identical to those present in the standard; however compounds identical to the putative additional antimycins present in the OM-WT extract were detected. The extracts from the SM8*-*Δ*ant*C strain were found to contain no traces of antimycin-like compounds. The chromatograms are shown in Figure 10, a summary of the antimycin metabolites identified is shown in Table 4, and the structures of antimycins A1–A4 together with the fragmentation of the dilactone ring of antimycin A1a to generate the fragment ion with an *m/z* of 265.081 are shown in Figure 11. Using a single point calibration assay, total antimycin content can be quantified from the summation of peak areas of extracted ion peaks of detected antimycin congeners in comparison to 1 mg/mL concentration of standard antimycin mixture sample. OM-WT and SYP-WT extract had a total antimycin content of 161.61 and 15.10 μg/mL, respectively as based on peaks exhibiting a fragment ion at *m/z* 265.081.

**Table 4:** Identification of antimycins in the SM8 extracts. Compounds were identified as antimycins based on comparison to authentic antimycin standards (STD). Compounds with identical *m*/*z* values and based on the presence of the characteristic fragment ion at *m/z* 265.081 [M + H]^+^ (indicated by **+**) were putatively identified as antimycins. n.d (*m/z* 265 not detected).

Antimycin A	*m*/*z* [M + H]^+^	STD		OM WT		OM *ant*C		SYP WT		SYP *ant*C	
		RT	*m/z* 265	RT	*m/z* 265	RT	*m/z* 265	RT	*m/z* 265	RT	*m/z* 265
1	549.280			22.54	+		n.d	22.56	+		n.d
		27.92	+	27.95	+		n.d				n.d
2	535.264			19.97	+		n.d	20.90	+		n.d
				21.26	+		n.d	21.27	+		n.d
		25.61	+	25.23	+		n.d				n.d
		26.58	+	26.58	+		n.d				n.d
3	521.249			19.77	+		n.d	19.65	+		n.d
		23.95	+	23.68	+		n.d				n.d
		25.08	+	25.07	+		n.d				n.d
4	507.233	23.58	+	23.59	+		n.d				n.d
5	493.217	22.27	+		n.d		n.d				n.d
6	479.202	20.35	+		n.d		n.d				n.d
		20.72	+		n.d		n.d				n.d

**Table:** 

Antimycin	R_1_	R_2_
A1a	C=OCH(CH_3_)CH_2_CH_3_	(CH_2_)_5_CH_3_
A1b	C=OCH_2_CH(CH_3_)_2_	(CH_2_)_5_CH_3_
A2a	C=OCH(CH_3_)_2_	(CH_2_)_5_CH_3_
A2b	C=OCH_2_CH_2_CH_3_	(CH_2_)_5_CH_3_
A3a	C=OCH(CH_3_)CH_2_CH_3_	(CH_2_)_3_CH_3_
A3b	C=OCH_2_CH(CH_3_)_2_	(CH_2_)_3_CH_3_
A4a	C=OCH(CH_3_)_2_	(CH_2_)_3_CH_3_
A4b	C=OCH_2_CH_2_CH_3_	(CH_2_)_3_CH_3_
Deisovaleryl antimycin A3	H	(CH_2_)_3_CH_3_
A5	C=OCH_2_CH(CH_3_)_2_	CH_2_CH_3_
A6	C=OCH_2_CH_2_CH_3_	CH_2_CH_3_

Antimycin A was first isolated from an unknown soil *Streptomyces* sp. [30]. It was discovered to be a mixture of four components, named A1 to A4, which were then purified and characterized. Antimycin A3 was found to be the most active component [31,32] although this appears to be due to its diffusion coefficient in agar [33]. Since then, many other compounds falling under the antimycin A family have been isolated from other *Streptomyces* species [34]. These compounds possess antifungal activity and act by blocking the electron transport chain via inhibition of the cytochrome *bc*_1_ complex. Gram positive bacteria lack this antimycin sensitive site and hence are unaffected by antimycin A [35]. In the course of this study it was noted that the *antC* mutant strain had reduced antibacterial activity against *P. aeruginosa* PAO1, but not against any of the other bacterial strains tested. This was investigated further and it was discovered that the purified antimycin standard mixture had a previously unreported activity antibacterial activity against *P. aeruginosa* PAO1 with an MIC of 0.05 mg·mL^−1^. Aside from being a well-known antifungal, antimycin A is also a fish toxicant [36], and therefore could be of value to a sponge when produced by an endosymbiont.

The optimum conditions for antimycin production have previously been studied in *S. antibioticus*. Antimycin production in *S. antibioticus* has been shown to increase when the tryptophan concentration in the media was increased to 2 mg·mL^−1^ after which there was decreased antimycin production. It has been proven that the benzene ring of dl-tryptophan is incorporated into antimycin A [37]. More recent studies have shown that tryptophan is the precursor from which the 3-formamidosalicylic acid (FSA) moiety is formed. The dilactone core is then created by sequential elongation with threonine and pyruvate, followed by cyclization to close the ring [38]. In this study, tryptophan was present in the yeast extract that was a component of the SYP-SW media, but to what extent this affected antimycin A production in SM8 is unknown. OM-SW media was found to be optimum for antimycin A production, however tryptophan was not found in the mass spectrum of the blank oatmeal media. The regulation of expression of antimycin gene clusters is complex, involving a pathway specific sigma factor, present in the gene cluster (*ant*A) which controls genes for the production of the FSA moiety with as yet unknown factors controlling expression of the other biosynthesis genes [39]. While OM-SW media is certainly the media of choice for antimycin production, other media, such as SYP-SW may be preferred for isolation of antifungal compounds other than the antimycins. These results highlight the advantages of using both genomics and metabolomics as complementary techniques. The presence of the antimycin biosynthetic gene cluster in the SM8 genome facilitated the identification of these compounds in the extracts and metabolomic analyses have demonstrated the differences in the production of metabolites depending on the growth conditions used. Future work will correlate gene expression and metabolomics data.

#### 2.2.3. Isolation of Compounds from Large-Scale Cultures of *Streptomyces* SM8

A larger scale culture of SM8 grown on oatmeal media was extracted in order to isolate compounds. Although the antimycins were not obtained as pure compounds, LC-HRMS data indicated that the presence of antimycins in the fractions correlated with antifungal activity.

**Figure 12:**
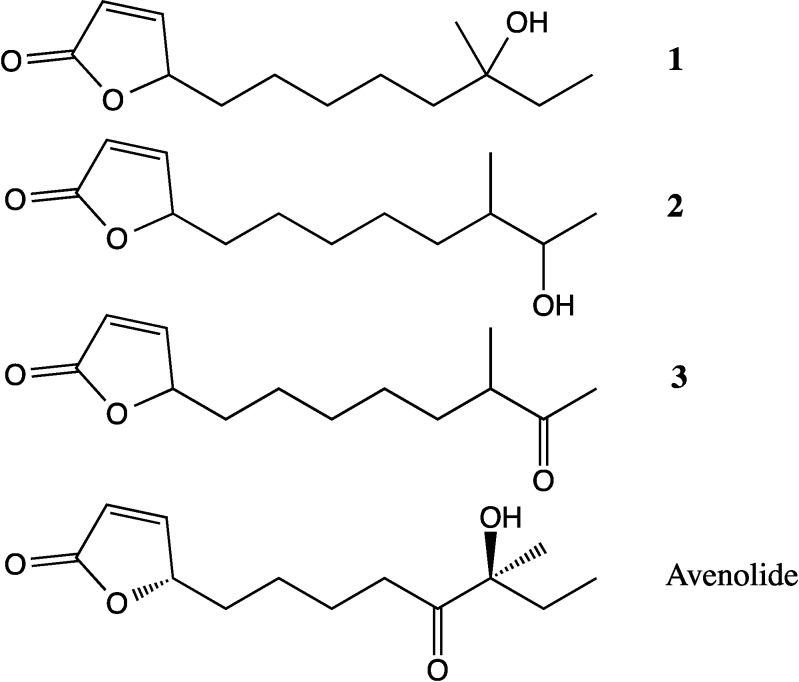
Butenolides isolated from SM8 (**1**–**3**) and avenolide, a butenolide produced by *S. avermitilis*.

Three butenolides (Figure 12) were obtained that had previously been isolated from marine *Streptomyces* sp. [40]. These were identified as 4,10-dihydroxy-10-methyl-dodec-2-en-1,4-olide (**1**), 4,11-dihydroxy-10-methyl-dodec-2-en-1,4-olide (**2**), and 4-hydroxy-10-methyl-11-oxo-dodec-2-en-1,4-olide (**3**). The structures were confirmed using HRESIMS and NMR.

Butenolide **1** has also been isolated from a *Streptomyces* sp. cultivated from a Mediterranean sponge *Tethya* sp. and has been proven to have inhibitory activity against *Trypanosoma brucei brucei* [41]. These butenolides were also present in the antifungal fractions, although the antifungal activity can be attributed to the antimycins that were likewise present in the fractions as purified fractions of these butenolides were found to lack antifungal activity. Some furanones have quorum signaling activity [42] and it is possible that these butenolides synthesized by SM8 play a regulatory role. The butenolide avenolide (Figure 12) has been shown to have a role in the regulation of antibiotic production in *S. avermitilis* [43]. Butenolides are similar in structure to the γ-butyrolactones, the main differences being the position of the side chain, the presence of a double bond in the butenolides, and the presence of an alcohol functional group on the γ-butyrolactones. γ-Butyrolactones are common signaling molecules in *Streptomyces*, with analogous functions to *N*-acyl homoserine lactone (AHL) signaling molecules of Gram-negative bacteria [44], and the butenolide avenolide has been shown to interact with regulatory proteins that are closely related to those involved in regulation via γ-butyrolactones. In the avenolide-producing organism *S. avermitilis*, a proposed biosynthetic gene cluster was identified consisting of a regulatory gene, a cytochrome P450 hydroxylase gene and an acyl-CoA oxidase gene. The same arrangement of genes was not present in the SM8 genome, and at present the biosynthetic pathway of this class of compounds is unknown. Butenolides have also been isolated from sponges [45] and research has shown that furanones may be used by eukaryotic host organisms to interfere with prokaryotic signaling, thereby regulating the colonies they play host to [46,47].

Volatile butenolides and other furanone derivatives have previously been identified as products of antimycin degradation [48] and thus were not found in *Streptomyces* mutants lacking the antimycin gene cluster. The three butenolides isolated from SM8 differed from these degradation products in that, unlike the volatile butenolides, they lacked the 2-alkyl group and possessed an alkyl chain ratherthan a methyl group on C-4. These compounds evidently arose from a different biosynthetic pathway unrelated to the antimycin pathway as their production was not diminished in the Δ*ant*C mutant.

### 2.3. Comparison of Endosymbiont and Host Sponge Metabolic Profiles

#### 2.3.1. LC-HRESIMS Comparison

**Figure 13:**
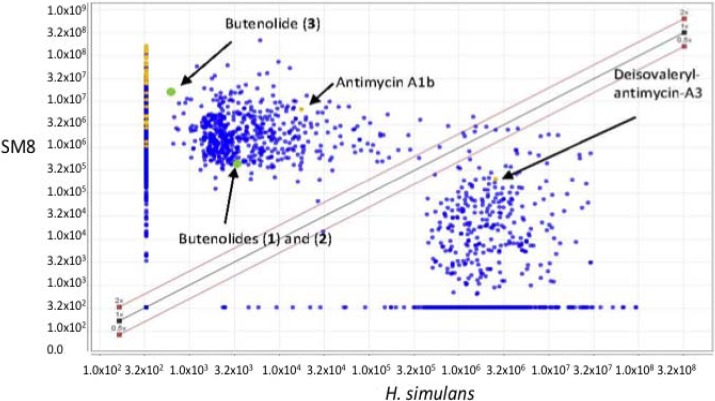
Scatter plot comparison of *H. simulans* (*x*-axis) and SM8 (*y*-axis) in the positive ionization mode. Metabolites identified as belonging to the antimycin family of compounds are highlighted in yellow whereas the isolated butenolides are highlighted in green. Although the differences in concentration are to be expected, the presence of common metabolites in the extracts confirmed that some metabolites in the *H. simulans* extract are products of its symbiont, SM8.

The LC-HRESIMS spectra of the *Streptomyces* SM8 and *H. simulans* extracts, both prepared as 1 mg·mL^−1^ in 70:30 methanol:dichloromethane were processed using a customized version of MZmine 2.10 [49]. After peak picking, alignment of the peaks allowed the generation of a scatter plot (Figure 13) to compare the metabolites present in each extract. Metabolites falling within the diagonal are present in approximately the same concentration in both extracts whereas those outside the diagonal are more prominent in the sample stated in the corresponding axis. Most antimycin compounds were found only in the SM8 extract, which is unsurprising as antimycins are bacterial products. However, antimycin A1b was detected in the sponge extract, albeit at a lower intensity than in the SM8 extract. Deisovaleryl-antimycin A3 (Figure 11) was found in higher concentrations in the sponge extract than the bacterial extract. From our studies it is clear that *Streptomyces* strain SM8 has the capacity to produce different antimycin metabolites in different conditions and it is possible that the growth conditions in the sponge favored the production of this particular antimycin. It is also possible that the isovaleryl side group is removed from the antimycin as a result of sponge metabolism, resulting in the high level of this compound in the *H. simulans* extract. The butenolides **1** and **2** were present in low quantities in the sponge extract, but butenolide **3** was found solely in the SM8 sample.

**Figure 14:**
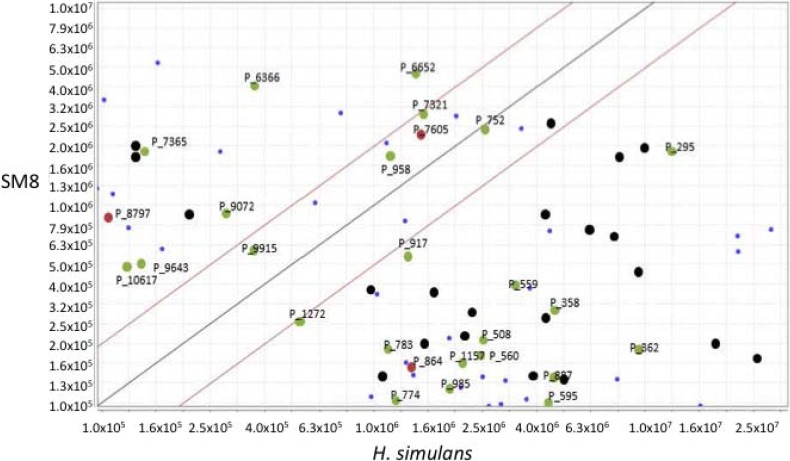
Common metabolites in *H. simulans* (*x*-axis) and *Streptomyces* SM8 (*y*-axis) extracts with peak areas >1.0 × 10^5^ in the positive ionization mode. Blue circles are unidentified; black circles are metabolites previously isolated from fungi, algae and other marine invertebrates; green circles are those previously isolated from sponges and/or bacteria; red circles are those isolated from *Haliclona* sp.

Dereplication of the other metabolites common to both samples resulted in highlighting compounds isolated from a variety of sources, including sponges, bacteria, fungi, algae and other marine invertebrates. Figure 14 shows the expansion of the scatter plot in the ESI positive mode with the focus on compounds that are present in both samples and have a peak area greater than 1.0 × 10^5^. No peaks with this peak area fell within the diagonal in the ESI negative mode. The metabolites are colored according to their source. The identified metabolites falling within the diagonal can be seen in Table 5. The majority of these compounds have previously been isolated from *Streptomyces* sp. Thus, although these compounds were not isolated from SM8, the fact that these metabolites are produced by the same genus gives credence to their putative identifications.

The scatter plot illustrates that there are metabolites which are common between the sponge and its endosymbiont. This highlights the relationship between the two organisms; that is, it confirms that *Streptomyces* SM8 is likely to be a true symbiont of *H. simulans*. Several of the putative compounds in Table 5 above are small MW peptides and are possibly produced by one of the NRPS gene clusters present in *Streptomyces* SM8, while the compound with Mol_ID P 9915 (9-(4-aminophenyl)-7-hydroxy-2,4,6-trimethyl-9-oxo-non-2-enoic acid) is a truncated version of candicidin that could have arisen as a shunt product of the candicidin pathway or as a degradation product. Although a candicidin gene cluster was present in *Streptomyces* SM8, intact candicidin was not detected in any of the culture broths. It is possible that some metabolites produced by *Streptomyces* SM8 are utilized by *H. simulans* for defensive or regulatory purposes; as previously mentioned, antimycins can act as both antifungal and fish deterrent compounds, and the butenolides may function as signaling molecules.

**Table 5:** Common metabolites present in the extracts of *H. simulans* and *Streptomyces* SM8 (positive ionization). The compounds were putatively identified using the AntiMarin 2012 database. Only those from sponges or bacteria are included in the table.

Mol_ID	*m/z*	RT (min)	Formula	Name	Source
P_752	295.1649	4.00	C_15_H_22_N_2_O_4_	1328-3; Cyclocarbamide B	[B] *Streptoverticillium* sp.
P_7321	295.1641			(2*S*,3*R*)-3-Amino-2-hydroxy-1-phenylbutanoyl-l-valine	[B] *Streptomyces neyagawaensis* SL-387 + dl-3-amino-3-phenylpropionic
P_7605	206.0812	6.62	C_11_H_11_NO_3_	Indole-3-lactic acid	[B] *Streptomyces* sp. L083; marine *Streptomyces* sp. 7919
				1-(3-Indolyl)-2,3-dihydroxypropan-1-one	[B] *Streptomyces violaceus*, marine
				1-Hydroxymethyl-7-methoxyisoquinolinol	Porifera *Haliclona* sp
P_958	243.1338	5.27	C_11_H_18_N_2_O_4_	Bicycloamid;	[B] *Streptomyces albus* Tue 2031
				3,7-Dihydroxy-*cis*,*cis*-1,8-nonadiene-1,9-dicarboxylic acid diamide	[B] marine actinomycete B 1758
					Porifera *Asteropus* sp.
P_9915	320.1852	14.93	C_18_H_25_NO_4_	9-(4-Aminophenyl)-7-hydroxy-2,4,6-trimethyl-9-oxo-non-2-enoic acid	[B] *Streptomyces griseus* subsp.
					[B] *Streptomyces* sp

#### 2.3.2. GC-MS Comparison

**Figure 15:**
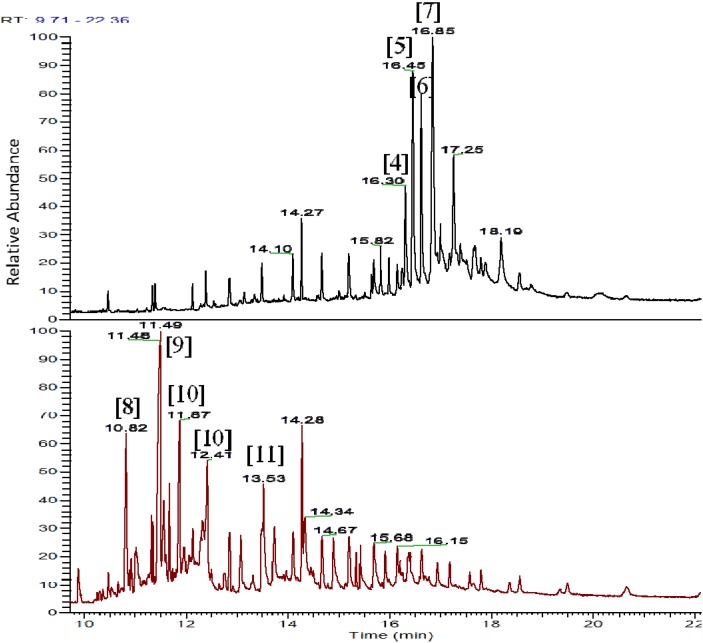
GC-MS chromatograms of *H. simulans* (**top**) and *Streptomyces* SM8 (**bottom**).

The ethyl acetate-soluble components of *H. simulans* and *Streptomyces* SM8 were compared using GC-MS. For dereplication purposes as well as to exhaust all information about the investigated extracts, GCMS data also was collected in parallel to those found by LC-HRESIMS. In this case, the results can be compared taking advantage of the capability of each ionization technique in relation to the chemistry of the compounds being detected. Figure 15 shows the GC-MS chromatograms of the two samples. It is evident that the sponge has peaks that elute later from the column. These compounds were most likely less volatile and thus require a higher temperature to elute.

The GC-MS peaks were dereplicated using the online NIST11 library. All those present in the *H. simulans* sample at 15–17 min were all detected to be steroids. Through their respective EI (electron impact) mass spectrum, identified compounds dereplicated from the library had matching factor scores ≥800 for the SI (similarity index) and RSI (reversed search index) while the (Prob) probability score were ≥70. Although three bioactive steroids were earlier isolated from *H. simulans* [50], the four identified steroids **4**, **5**, **6**, and **7** were not among those previously isolated. This indicated that steroids **4**, **5**, **6**, and **7** may be present in the sponge in smaller quantities. All four have previously been found in marine sources including sponges. The previously isolated steroids had short side chains and were more polar; thus they may not be volatile enough to be detected by GC-MS without derivatization. The elution capability was also influenced by the structural features such as the number of carbons on the side chain, the degree of saturation and location of double bonds in the cyclopentanoperhydrophenanthrene ring and/or in the side chain whether their orientation is *cis* or *trans*, and the number of methyl groups on C-4 [51,52,53]. For example, sterols with an ethyl group on C-24 in the side chain as in stigmasta-5,24(28)-dien-3-ol (**7**) had longer retention times than those with a methyl group as in ergosta-5,22-dien-3-ol (**6**). In addition, sterols with a double bond on C-22 elute earlier as found in cholesta-5,22-dien-3-ol (**4**) than sterols with no double bonds in the side chain like cholesterol (**5**). The elution order thus observed was: cholesta-5,22-dien-3-ol (**4**) < cholesterol (**5**) < ergosta-5,22-dien-3-ol (**6**) < stigmasta-5,24(28)-dien-3-ol (**7**).

**Figure 16:**
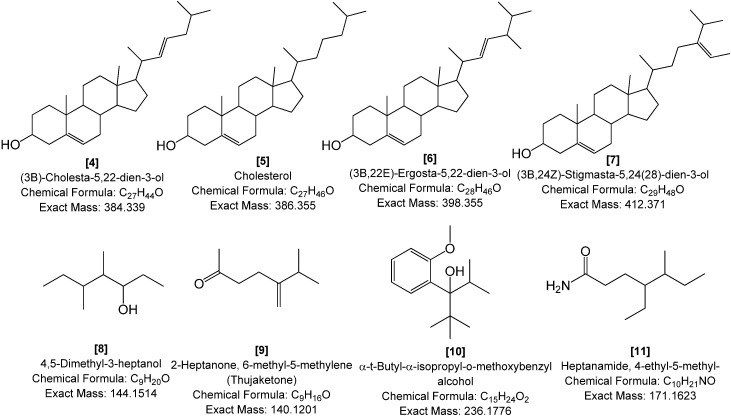
Metabolites putatively dereplicated from the *H. simulans* and *Streptomyces* SM8 extracts using the NIST11 GC-MS libraries.

In contrast to the sponge, the *Streptomyces* SM8 extract contained smaller molecules that had retention times of 10–13 min (Figure 15 and Figure 16). However, the dereplicated “hits” (**8**, **9**, **10**, and **11**) for the bacterial extracts gave poor matching scores, although the RSI factor were ≥800, the SI factors were ranging between 200 and 600 while the probability scores were less than 30. Among these “hits” were thujaketone (**9**) and 4-ethyl-5-methyl-heptanamide (**11**). Thujaketone (**9**) is commonly obtained from plants. Conversely, its carbon skeleton is the same as the side chain of ergosterol [54]. 4-Ethyl-5-methyl-heptanamide (**11**) has previously been isolated from a terrestrial *Streptomyces* sp. [55] as well as from freshwater green alga [56]. The MS spectrum for this compound, a fatty acid amide, contained a peak at *m*/*z* 59 that is characteristic of aliphatic primary amides [56].

## 3. Experimental Section

### 3.1. Culture of Streptomyces Strain SM8

*Streptomyces* strain SM8 was isolated from the marine sponge *Haliclona simulans* as described previously [8]. For preparation of extracts, seed cultures (50 mL broth in 250 mL conical flasks) of 410-SW broth (modified from [57]) (10 g glucose, 10 g glycerol, 15 g casamino acids, 5 g oatmeal, 10 g peptone, 5 g yeast extract, 1 g calcium carbonate, 33.3 g Instant Ocean^®^ per liter of dH_2_O) were inoculated with spores and incubated for 7 days at 28 °C with shaking at 200 rpm. These seed cultures were used to inoculate production flasks (50 mL broth in 250 mL conical flasks or 800 mL broth in 2 L conical flasks) containing OM-SW broth (modified from [55]) (20 g oatmeal, 33.3 g Instant Ocean^®^ per liter of dH_2_O) or SYP-SW broth (10 g starch, 4 g yeast extract, 2 g peptone, 33.3 g Instant Ocean^®^ per liter of dH_2_O) at a 1:50 dilution and incubated for 12 days at 28 °C in a shaking incubator at 200 rpm. All extracts were prepared in triplicates.

### 3.2. Preparation of Active Crude Extracts of Streptomyces Strain SM8

A total of 16 L of the culture broth was clarified by centrifugation at 12,000× *g* for 15 min in a Beckmann Coulter Avanti J-26 centrifuge (Pasadena, CA, USA). The supernatant was passed through Miracloth (Calbiochem, San Diego, CA, USA) to remove the debris. Initial compound extraction was performed by adding 70 g of prewashed amberlite XAD-16 per liter of culture and incubating overnight with shaking. The resin was collected by filtration and the extraction was repeated. Active compounds were then eluted from the resin using an equal volume of methanol with stirring for 2 h. The methanol washing step was repeated several times until the eluate was colorless. The extract was then concentrated by evaporation and desalted by liquid-liquid partitioning with ethyl acetate and water. The ethyl acetate fraction was then evaporated to dryness. All extracts were prepared in triplicates.

### 3.3. Bioassays

Crude extracts and purified fractions in triplicates were assayed for antimicrobial activity using the following indicator strains: *Candida albicans* SC5314, *Candida glabrata* CBS138, *Saccharomyces cerevisiae* BY4741, *Kluyveromyces marxianus* CBS6556, *Aspergillus fumigatus* Af293, *Bacillus subtilis* IE32, *Escherichia coli* 12210, *Staphylococcus aureus* NC000949 and *Pseudomonas aeruginosa* PA01. Antifungal [58] and antibacterial [59] activities were determined using resazurin based assays performed with dilution series of extracts in 96 well microtiter plates.

### 3.4. Genomic Sequencing

#### 3.4.1. Extraction of Genomic DNA

*Streptomyces* strain SM8 was grown in SYP-SW broth for three days at 28 °C in a shaker incubator at 200 rpm and the genomic DNA was extracted using the cetyltrimethylammonium bromide (CTAB) method [60]. DNA was quantified using a NanoDrop spectrophotometer (Thermo Scientific, Bremen, Germany) and analyzed by agarose gel electrophoresis. The concentration of the DNA was adjusted to 200 ng·μL^−1^ for genome sequencing.

#### 3.4.2. Genome Sequencing and Analyses

Genomic sequencing was carried out by the Centre for Genomic Research, University of Liverpool. The nucleotide sequence was generated from a fragment library using the GS FLX Titanium system (Roche 454 Sequencing, Branford, CT, USA) resulting in 229,280 reads and 94,668,678 bp, giving approximately 10-fold coverage of the genome. The genome was assembled using the Meta-assembler program hosted by Community cyberinfrastructure for Advanced Microbial Ecology Research and Analysis (CAMERA 2.0) [61] and the reads were quality filtered resulting in 539 contigs. The genome was annotated using the Prodigal pipeline hosted by IMG/ER [62]. Secondary metabolite genes were additionally identified using antiSMASH [63] that rapidly identifies a whole range of known secondary metabolite compound classes. Contigs identified as containing secondary metabolism genes were used as queries to scan for functional similarities both in Clusters of Orthologous Groups of proteins (COGs) and Pfam databases in IMG/ER. The IMG/ER has about 88 COG proteins and 52 Protein families (Pfam) relating to secondary metabolism and synthesis, catabolism and transport. The protein sequences corresponding to the secondary metabolite genes were searched in BLASTp [64]. The sequences were also compared to other *Streptomyces* species in the NCBI database using BLAST to identify the closest homologue. The genome sequence is deposited at GenBank with accession number PRJNA180938.

### 3.5. Identification and Isolation of Bioactive Metabolites of SM8

#### 3.5.1. Reverse Phase MPLC of Small Scale SM8 Extract

The SM8 extract, weighing 343.7 mg, was subjected to medium pressure liquid chromatography (MPLC) using a Sepacore^®^ chromatography system (Büchi Labortechnik, Basel, Switzerland) consisting of two C 605 pump modules, a C 620 control unit over a VersaPak C-18 (Spherical) 23 × 53 mm (15 g) column. The column was eluted with water (A) and methanol (B) using the following stepwise gradient: 1% B (5 min), 1%–50% B (30 min), 50% B (5 min), 50%–100% B (25 min). The flow rate was 10 mL·min^−1^. Fractions were collected every 15 s. A total of 264 fractions were obtained. These were pooled according to their LC-MS chromatograms as measured by the low resolution Finnigan LCQ-Deca mass spectrometer coupled to an HPLC (series 1100) from Agilent (Santa Clara, CA, USA).

#### 3.5.2. NMR and LC-HRESIMS of Active SM8 Fractions

Following activity assays, the active fractions were analyzed by NMR using a Jeol-LA400 FT-NMR spectrometer system (JEOL Ltd., Tokyo, Japan) with an AS400 magnet (Oxford Instruments, Inghilterra, UK) at 400 MHz for ^1^H and 100 MHz for ^13^C, using a Pulse Field Gradient “Autotune” 40TH5AT/FG broadband high sensitivity probe (JEOL Ltd., Tokyo, Japan) to accept 5 mm tubes. High-resolution mass spectrometry was carried out on a Dionex UltiMate-3000 (DIONEX, Sunnyvale, CA, USA) coupled to an Exactive-Orbitrap (Thermo Scientific, Bremen, Germany). The column was an ACE 5 C18 75 × 3.0 mm column from Hichrom Ltd, Reading, UK. Compounds were eluted with a flow rate of 300 μL·min^−1^ using water (A) and acetonitrile (B), both of which contained 0.1% formic acid, by a gradient starting with 10% B and increasing to 100% B in 30 min. The mobile phase was maintained at 100% B for 5 min, after which the column was equilibrated with 10% B. The injection volume was 10 μL and the tray temperature was maintained at 12 °C. High resolution mass spectrometry was carried out in both positive and negative ESI ionization modes with a spray voltage of 4.5 kV and capillary temperature of 268 °C. Spray voltage at 4.5 kV, spray current set 10.67 μA, and capillary voltage at 30 V. The mass range was acquired from *m/z* 150 to 1500.

Multi-fragmentation (MS*^n^*) experiments were accomplished for the positive ionization mode on an Orbitrap analyser, CID (The collision-induced dissociation) was utilized with a normalized collision energy of 35%, activation Q of 0.250 ms, and activation time of 30.000 ms applied on ions of most intense, 2nd most intense, and 3rd most intense peaks for MS2, MS3, and MS4, respectively at an isolation width of 3 microns with 5 microscans. Resolution was at 15,000 m/Δm 50%, while the minimum ion signal threshold was set to 500. Fragment mass tolerance for molecular formula detection was set to ±5 ppm.

Data mining was performed using MZmine 2.10 [49]. The following parameters were used:

The chromatograms were first cropped to 0.5–38.0 min using the crop filter under the dataset filtering function. The centroid mass detector was used for peak detection with the noise level set to 1.0 × 10^5^ and the MS level set to 1. The chromatogram builder function was set to a minimum time span of 0.2 min, minimum height of 1.0 × 10^5^ and *m/z* tolerance of 0.001 *m/z* or 5.0 ppm. For chromatogram deconvolution the algorithm used was the local minimum search. The chromatographic threshold was set to 90.0%. The search minimum in RT range was 0.4 min, minimum relative height was 5.0%, minimum absolute height was 3.0 × 10^5^, minimum ratio of peak top/edge was 2 and the peak duration range was 0.3–5.0 min. Isotopes were detected using the isotopic peaks grouper. The *m/z* tolerance was 0.001 *m/z* or 5.0 ppm, RT tolerance was 0.2 absolute (min), the maximum charge was 2 and the representative isotope used was the most intense. Retention time normalization was performed using the RT normalizer. Again, *m/z* tolerance was 0.001 *m/z* or 5.0 ppm while the RT tolerance and the minimum standard intensity were set to 5% (relative) and 5.0 × 10^3^ respectively. The peak lists were all aligned using the join aligner (*m/z* tolerance 0.001 *m/z* or 5.0 ppm, weight for *m/z*: 20, RT tolerance: 5.0% relative, weight for RT: 20). The aligned peak list was gapfilled using the peak finder function (intensity tolerance: 1%, *m/z* tolerance: 0.001 *m/z* or 5.0 ppm, RT tolerance: 0.5 min). An adduct search was performed with the RT tolerance set at 0.2 absolute (min), the *m/z* tolerance at 0.001 *m/z* or 5.0 ppm and the maximum relative adduct peak height at 30%. The adducts searched for were Na^+^, K^+^, NH_4_^+^ and ACN + H. A complex search was also performed using [M + H]^+^ for the ESI positive mode and [M − H]^−^ for the ESI negative mode. The RT tolerance was set at 0.2 absolute (min), *m/z* tolerance was kept at 0.001 *m/z* or 5.0 ppm, and the maximum complex peak height was set at 50%. A custom database search was then performed using the DNP 2012 database [65].

#### 3.5.3. Construction of SM8 *ant*C Mutant

To inactivate the antimycin gene cluster, a two-step gene deletion procedure was used [60]. Flanking regions from within the *ant*C gene of approximately 1.4 kb were amplified using the High-Fidelity PCR kit (Roche, Basel, Switzerland). Primer pairs (Table 6) were designed to amplify upstream and downstream regions with restriction sites engineered suitable for cloning into the *Streptomyces/E. coli* shuttle vector pKC1139 [66]. PCR products from *ant*C1-F/*ant*C1-R and *ant*C2-F/*ant*C2-R were cloned into pJET1.2/blunt using the Fermentas PCR cloning kit. Following sequence verification the *ant*C flanking regions were purified as *Hin*dIII-*Xba*I and *Xba*I-*Eco*RI fragments, respectively and cloned into *Hin*dIII and *Eco*RI digested pKC1139 resulting in pKC1139A1A2.

**Table 6:** Primers designed for the construction of the *ant*C deletion strain. Engineered restriction sites are shown underlined.

	Primer Sequences (5′–3′)
*ant*C1-F	TATATAAAGCTTGGACGGCTACAGCTACAAGC
*ant*C1-R	TATATATCTAGAATGAGGTATGCGGTGTCGTA
*ant*C2-F	TATATATCTAGAGAGGTGGTTCGTGGAGGAG
*ant*C2-R	TATATAGAATTCTGACGATGATGACGTCCTTG

pKC1139A1A2 was conjugated to *Streptomyces* sp. SM8 strain. Competent cells of the dam-/dcm-*E. coli* C2925/pUZ8002 were transformed to apramycin resistance with pKC1139A1A2. The helper plasmid pUZ8002 can supply transfer functions to oriT-carrying plasmids, such as pKC1139. Cultures of *E. coli* C2925/pUZ8002/pKC1139A1A2 were grown in 10 mL of LB containing 25 μg kanamycin/mL, 25 μg chloramphenicol/mL and 50 μg apramycin/mL overnight at 37 °C. The overnight culture was diluted (1:10) in LB containing the aforementioned antibiotics and grown at 37 °C to an OD600 of 0.5. The cells were washed twice with an equal volume of LB to remove antibiotics and resuspended in 0.1 vol. of LB. Approximately, 10^8^ spores of the SM8 strain were added to 500 μL of 2× YT broth (1.6% peptone, 1% yeast extract, and 0.5% NaCl) and subjected to heat shock at 50 °C for 10 min, 0.5 mL of *E. coli* cells were added and the spores were briefly mixed. The pellet was resuspended and plated onto MS-SW agar (mannitol 20 g, soya flour 20 g, agar 20 g, Instant Ocean^®^ 33 g made up to 1 L with dH_2_O). Following incubation at 28 °C for 20 h the plates were overlayered with 1 mL antibiotic solution containing 0.5 mg nalidixic acid and 2 mg apramycin. These plates were further incubated at 28 °C until potential exconjugants were observed. The transconjugants were plated on to SYP-SW media containing nalidixic acid 25 μg·mL^−1^ and apramycin 100 μg·mL^−1^ [60] and incubated at 28 °C until exconjugants were observed.

The shuttle vector pKC1139A1A2 has a (pSG5) temperature-sensitive origin of replication. In *Streptomyces* it can replicate only at temperatures between 28 and 30 °C. When potential exconjugants were plated on to SYP-SW containing apramycin 100 μg·mg^−1^ and incubated at 37 °C, the vector stops replicating and apramycin resistance is only maintained when the plasmid is integrated onto the chromosome by homologous recombination. Apramycin resistant recombinant clones were allowed to sporulate on SYP-SW agar at 37 °C. Spores were collected and plated out to single colonies at 28 °C without selection. Single colonies were then picked and screened for the loss of apramycin resistance, a phenotype indicating that the plasmid has been lost with deletion of the *ant*C gene.

The wild-type and mutant SM8 extracts were compared to an antimycin A standard (Antimycin A, from *Streptomyces* sp.) purchased from Sigma-Aldrich Co (St. Louis, MO, USA). The LC-HRMS analysis was performed on a Finnigan Surveyor system coupled to a Thermo-Finnigan LTQ Orbitrap system using the same column and method stated in section 3.5.2.

#### 3.5.4. Isolation of Compounds from Large Scale SM8 Culture

The large-scale SM8 extract, weighing 810.3 mg, was fractionated using a Sephadex^®^ LH20 column (Pharmacia, Stockholm, Sweden) with methanol as the mobile phase at a flow rate of 1 mL/15 min (0.067 mL·min^−1^) and a collection volume of 1 mL/test tube. 169 fractions were collected. An aliquot of each tube was subjected to activity assays. The active fractions were subsequently pooled based on their activity and similarities in the TLC.

The pooled fraction 127–156 displayed prominent antifungal activity. This fraction was further purified using a silica column with the following solvent systems: 95:5 hexane:ethyl acetate, 90:10 hexane:ethyl acetate, 80:20 hexane:ethyl acetate, and 50:50 hexane:ethyl acetate, followed by washing of the column with 70:30 dichloromethane:methanol, 50:50 acetone:methanol and 100% methanol. The fractions and purified compounds were subsequently subjected to activity assays and analyzed using NMR and LC-HRESIMS.

**4,10-Dihydroxy-10-methyl-dodec-2-en-1,4-olide (1)**: Rf 0.56 (80:20 MeOH:H_2_O, C-18 silica gel), blue-violet with anisaldehyde-sulfuric acid; UV_max_ 219.0 (MeOH); ^1^H NMR (400 MHz, DMSO) δ 7.84 (1 H, dd, *J* = 5.7, 1.4 Hz, H-3), 6.20 (1 H, dd, *J* = 5.7, 1.8 Hz, H-2), 5.15 (1 H, m, H-4), 1.71 (1 H, m, H-5a), 1.55 (1 H, dd, *J* = 14.3, 6.9 Hz, H-5b), 1.33 (2 H, q, *J* = 7.5 Hz, H_2_-11), 1.33 (2 H, H_2_-6), 1.28 (brd. s), 0.98 (3 H, s, CH_3_-13), 0.79 (3 H, t, *J* = 7.5 Hz, CH_3_-12); ^13^C NMR (100 MHz, DMSO) δ 173.6 (1-C=O), 159.3 (3-CH), 120.8 (2-CH), 83.7 (4-CH), 71.2 (10-C), 41.5 (9-CH_2_), 34.4 (5-CH_2_), 34.4 (11-CH_2_), 30.1 (7-CH_2_), 26.9 (13-CH_3_), 25.0 (6-CH_2_), 23.8 (8-CH_2_), 8.8 (12-CH_3_); HRESIMS: Calculated: *m/z* 227.1602 [M + H]^+^; Experimental: *m/z* 227.1637 [M + H]^+^.

**4,11-Dihydroxy-10-methyl-dodec-2-en-1,4-olide (2)**: Rf 0.56 (80:20 MeOH:H_2_O, C-18 silica gel), blue-violet with anisaldehyde-sulfuric acid; UV_max_ 219.0 (MeOH); ^1^H NMR (400 MHz, DMSO) δ 7.84 (1 H, dd, *J* = 5.7, 1.4 Hz, H-3), 6.20 (1 H, dd, *J* = 5.7, 1.8 Hz, H-2), 5.15 (1 H, m, H-4), 3.42 (2 H, m, H_2_-11), 1.71 (1 H, m, H-5a), 1.55 (1 H, dd, *J* = 14.3, 6.9 Hz, H-5b), 1.33 (2 H, H_2_-6), 1.28 (brd. s), 0.96 (3 H, d, *J* = 6.3 Hz, CH_3_-12), 0.77 (3 H, td *J* = 6.6 Hz, CH_3_-13); ^13^C NMR (100 MHz, DMSO) δ 173.6 (1-C=O), 159.3 (3-CH), 120.8 (2-CH), 83.7 (4-CH), 70.0 (11-CH_2_), 41.0 (10-CH), 34.4 (5-CH_2_), 32.8 (9-CH_2_), 30.1 (7-CH_2_), 27.2 (8-CH_2_), 25.0 (6-CH_2_), 19.9 (12-CH_3_), 15.1 (13-CH_3_); HRESIMS: Calculated: *m/z* 227.1602 [M + H]^+^; Experimental: *m/z* 227.1637 [M + H]^+^.

**4-Hydroxy-10-methyl-11-oxo-dodec-2-en-1,4-olide (3)**: UV_max_ 223.0 (MeOH); ^1^H NMR (400 MHz, DMSO) δ 7.84 (1 H, d, *J* = 5.9 Hz, H-3), 6.20 (1 H, dd, *J* = 5.6, 1.9 Hz, H-2), 5.14 (1 H, dd, *J* = 7.2, 5.1 Hz, H-4), 2.09 (3 H, s, CH_3_-12), 1.70 (1 H, m, H-5a), 1.55 (1 H, dd, *J* = 14.9, 7.4 Hz, H-5b), 1.27 (m), 0.98 (3 H, d, *J* = 6.9 Hz, CH_3_-13); HRESIMS: Calculated: *m/z* 225.1446 [M + H]^+^; Experimental: *m/z* 225.1486 [M + H]^+^.

### 3.6. Comparison of Endosymbiont and Host Sponge Metabolic Profiles

#### 3.6.1. LC-HRESIMS Analysis of Extracts

An acetone-methanol extract of *H. simulans* was de-salted with gradient elution through HP20 using water and methanol followed by 50:50 acetone:methanol and 100% methanol washes. An aliquot was taken from each fraction aside from the 100% water and the 90:10 aliquots, which contained the most salt. The pooled aliquot was dried down and resuspended in 10:90 H_2_O:MeOH to a final concentration of 1 mg·mL^−1^ for LC-MS. The SM8 sample was taken from the larger-scale extract and was dissolved in methanol. The samples were run on an Accela HPLC system coupled to a Thermo Scientific Exactive-Orbitrap using the same column and method stated in section 3.5.2. The results were compared using MZmine 2.10 [49].

#### 3.6.2. GC-MS Analysis of Extracts

The ethyl acetate-soluble portions of the extracts of *H. simulans* and SM8 were run on a Focus GC-DSQ II from Thermo Scientific (Thermo Fisher Scientific (Bremen) GmbH, Bremen, Germany). The column, InertCap 1MS (ID: 0.25 mm, length: 30 m, df: 0.25 μm) was from GL Sciences Inc., Tokyo, Japan. The oven temperature was set at 80 °C for 1 min after which the temperature was increased at a rate of 15° min^−1^ until 200 °C. This temperature was maintained for 15 min after which it was again increased at a rate of 5° min^−1^ until the final temperature of 320 °C. This was held for 10 min until the end of the run. The base temperature of the SSL was 250 °C whereas the MS transfer line was maintained at 320 °C. The electron ionization (EI) source temperature of the DSQ II mass spectrometer was set to 250 °C. The injection was performed in the splitless mode with helium as the constant carrier gas at a flow rate of 1.5 mL·min^−1^ and mass scan range at *m/z* 50.0–800.0.

## 4. Conclusions

The combined metabolomic and genomic approach used in this study resulted in the rapid identification of antimycins and polyhydroxylated saturated fatty acids as major antifungal and antibacterial metabolites of *Streptomyces* SM8, respectively. Bioassay-guided fractionation of large scale SM8 extracts also highlighted the presence of the antifungal antimycin family of compounds. Three butenolide compounds were also isolated and identified and while the biosynthetic origin of these compounds is currently unknown, the genome sequence of *Streptomyces* SM8 provides a genetic framework with which to study their biosynthesis and their potential role in regulation of gene expression. Likewise, as the SM8 Δ*ant*C mutant retained some antifungal activity, larger scale cultivation of these mutants can lead to the identification of potentially novel antifungal metabolites with genomic correlation to secondary metabolism gene clusters.

Comparison of the extracts of the host sponge *H. simulans* and its endosymbiont, *Streptomyces* SM8, using high resolution LC-MS revealed the presence of metabolites, including antimycins and a putative candicidin shunt or degradation product common to both extracts. This confirmed that SM8 is indeed associated with *H. simulans* and that bioactive components produced by *Streptomyces* SM8 are present in the sponge tissue, thus providing further evidence of the potential for microbial symbionts to provide their sponge host with a chemical defense system.

## Supplementary Files

Supplementary File 1 Supplementary Information (PDF, 447 KB)
