# Supplementary material for: Metabolomic Profiling and Genomic Study of a Marine Sponge-Associated Streptomyces sp
Source: Mar Drugs. 2014 Jun 2;12(6):3323–51. doi: 10.3390/md12063323 (PMC4071579; doi:10.3390/md12063323)
Supplement: Supplementary File 1 — Supplementary Information (PDF, 447 KB) [file marinedrugs-12-03323-s001.pdf]

Supplementary Information

Figure S1. Proton NMR of pooled fractions 221–230.

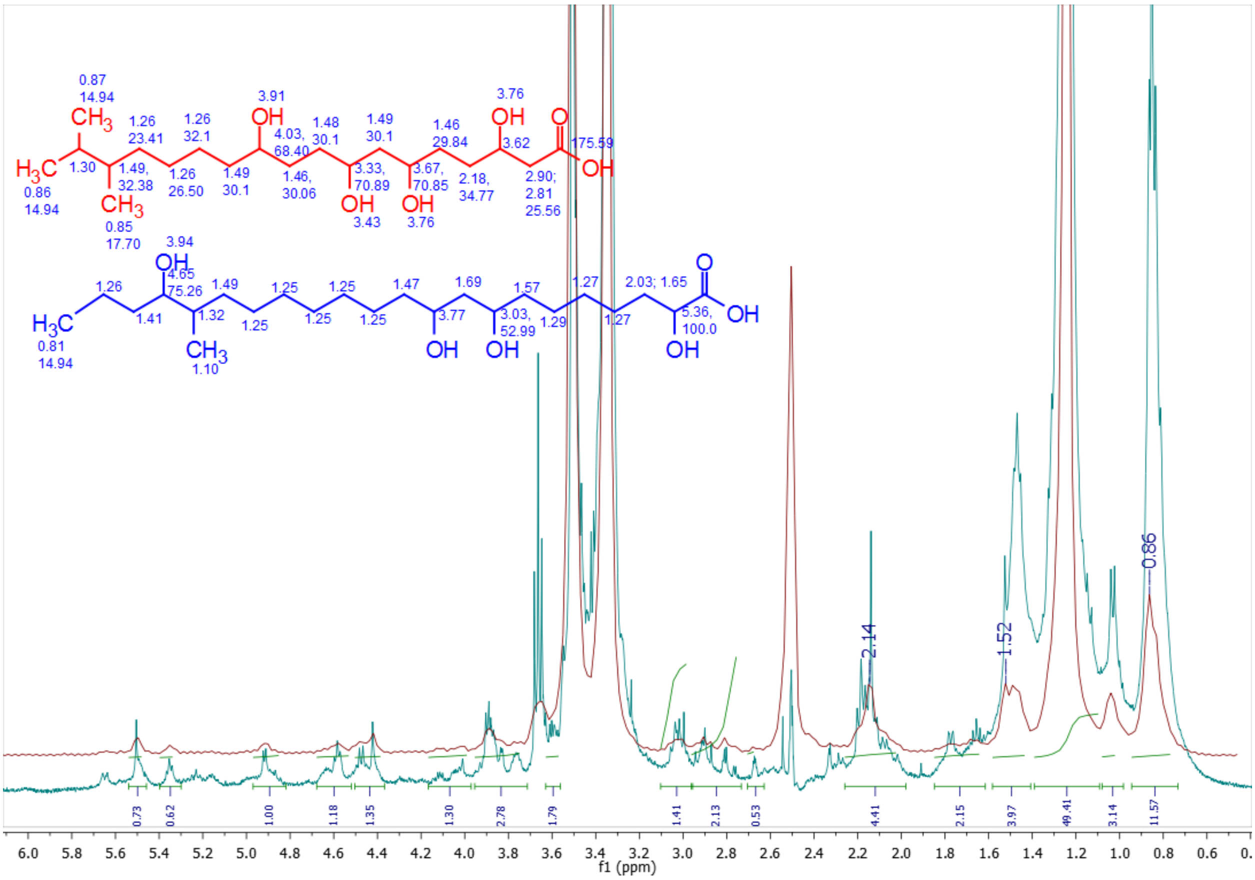

Figure S2. Carbon NMR of pooled fractions 221–230.

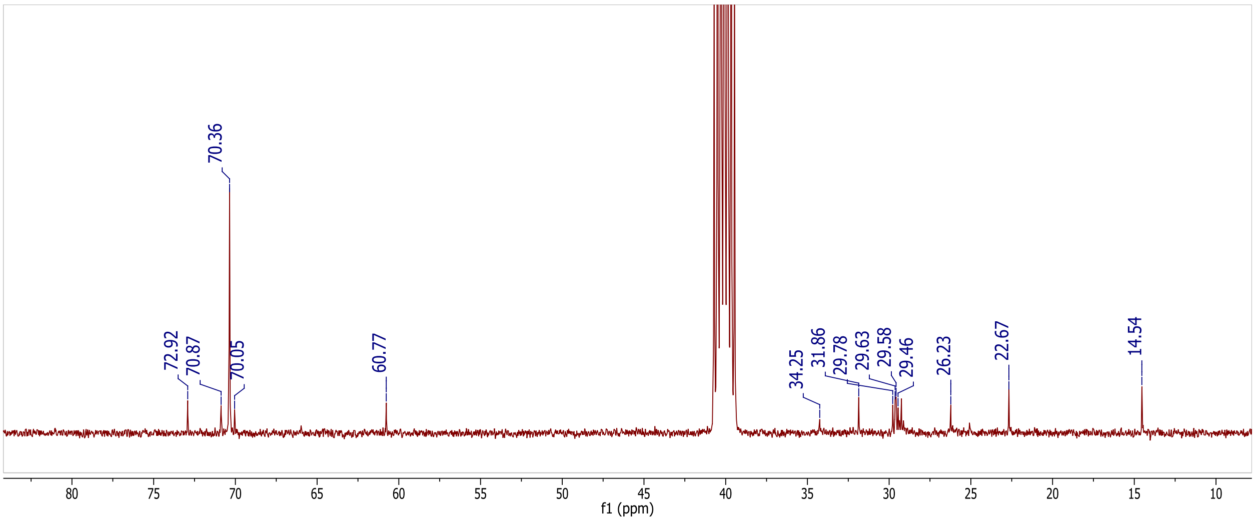

Figure S3. HMBC of pooled fractions 221–230.

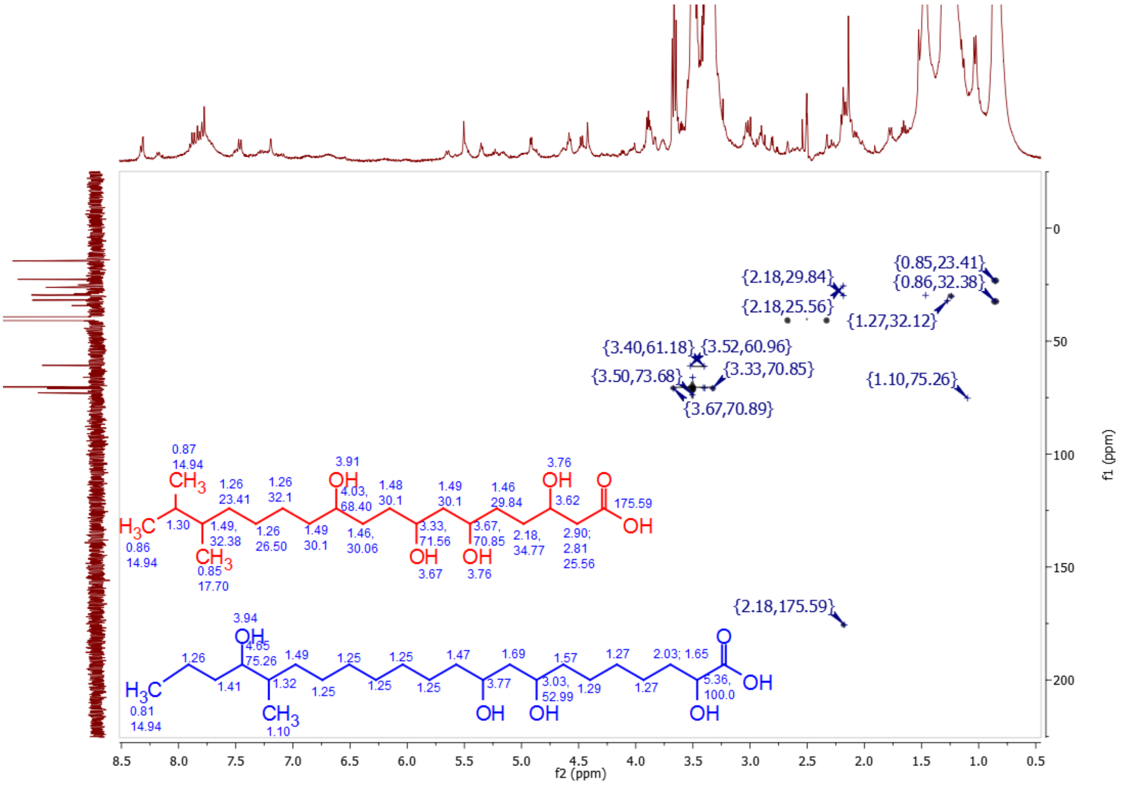

Figure S4. HMQC of pooled fractions 221–230.

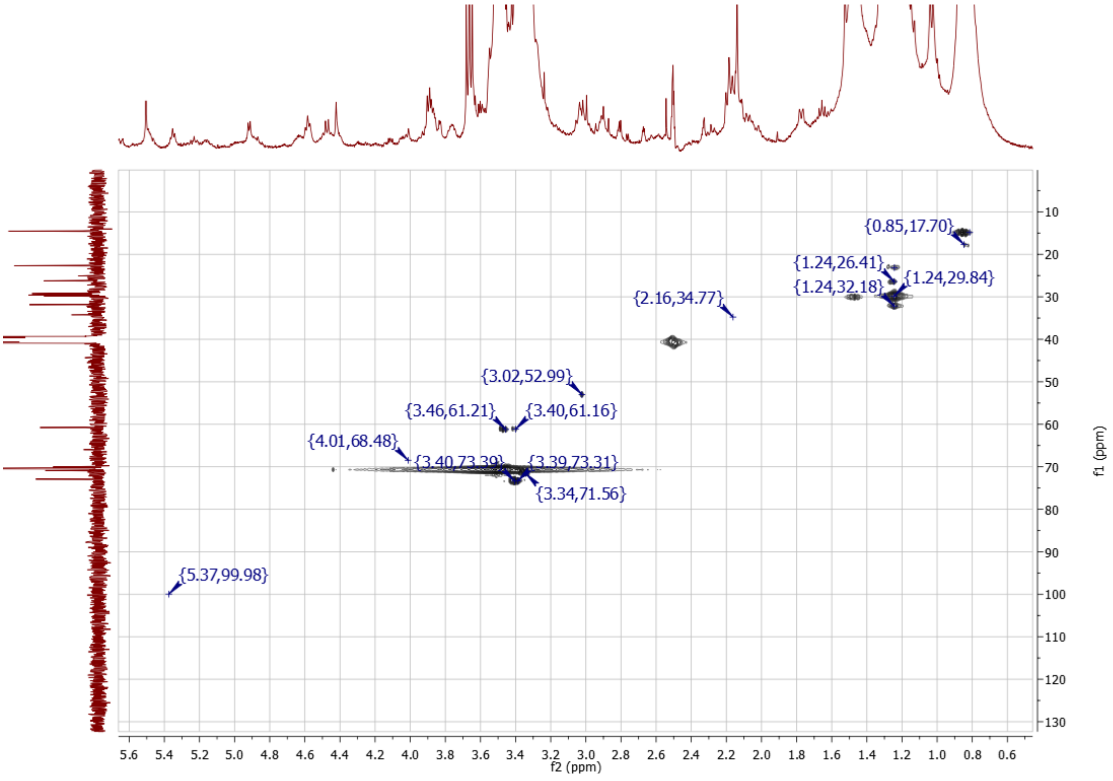

© 2014 by the authors; licensee MDPI, Basel, Switzerland. This article is an open access article distributed under the terms and conditions of the Creative Commons Attribution license (http://creativecommons.org/licenses/by/3.0/).
